# The Roles of RNase-L in Antimicrobial Immunity and the Cytoskeleton-Associated Innate Response

**DOI:** 10.3390/ijms17010074

**Published:** 2016-01-08

**Authors:** Heather J. Ezelle, Krishnamurthy Malathi, Bret A. Hassel

**Affiliations:** 1 Marlene and Stewart Greenebaum Cancer Center, University of Maryland School of Medicine, Baltimore, MD 21201, USA; hezelle@som.umaryland.edu; 2 Department of Microbiology and Immunology, University of Maryland School of Medicine, Baltimore, MD 21201, USA; 3 Research Services, Baltimore Veterans Affairs Medical Center, Baltimore, MD 21201, USA; 4 Department of Biological Sciences, University of Toledo, Toledo, OH 43606, USA; Malathi.Krishnamurthy@utoledo.edu

**Keywords:** RNase-L, innate immunity, cytoskeleton, actin, interferon, inflammasome, LNX, filamin A

## Abstract

The interferon (IFN)-regulated endoribonuclease RNase-L is involved in multiple aspects of the antimicrobial innate immune response. It is the terminal component of an RNA cleavage pathway in which dsRNA induces the production of RNase-L-activating 2-5A by the 2′-5′-oligoadenylate synthetase. The active nuclease then cleaves ssRNAs, both cellular and viral, leading to downregulation of their expression and the generation of small RNAs capable of activating retinoic acid-inducible gene-I (RIG-I)-like receptors or the nucleotide-binding oligomerization domain-like receptor 3 (NLRP3) inflammasome. This leads to IFNβ expression and IL-1β activation respectively, in addition to broader effects on immune cell function. RNase-L is also one of a growing number of innate immune components that interact with the cell cytoskeleton. It can bind to several cytoskeletal proteins, including filamin A, an actin-binding protein that collaborates with RNase-L to maintain the cellular barrier to viral entry. This antiviral activity is independent of catalytic function, a unique mechanism for RNase-L. We also describe here the interaction of RNase-L with the E3 ubiquitin ligase and scaffolding protein, ligand of nump protein X (LNX), a regulator of tight junction proteins. In order to better understand the significance and context of these novel binding partners in the antimicrobial response, other innate immune protein interactions with the cytoskeleton are also discussed.

## 1. Introduction

RNase-L is an endoribonuclease that cleaves single-stranded RNA as the effector portion of a two-component system regulated by the antiviral type I and III interferons (IFN-α/β and IFN-λ, respectively) [1,2]. IFN signaling leads to the induction of oligoadenylate synthetases (OAS) which, following activation by double stranded RNA (dsRNA), generate 2′-5′-linked oligoadenylates (2-5A) [3,4]. 2-5A then binds to RNase-L, leading to its dimerization and enabling its nuclease activity [4]. Although originally characterized as an antiviral agent, almost 40 years of research has unveiled many additional and diverse functions for this endoribonuclease, as well as novel mechanisms for both these new and its more established antimicrobial activities.

In the late 1970′s, the 2-5A-dependence and endonucleolytic properties of RNase-L were being investigated by multiple laboratories around the world [5,6,7,8,9]. The cloning of RNase-L by the Silverman lab in 1993 then opened the door for genetic manipulation and more definitive characterization of the mechanisms of RNase-L as a mediator of IFN activity, as well as identifying new physiologic roles [10]. Tumor suppressive activities, including antiproliferative and proapoptotic functions, were quickly ascribed and, in 2002, a genetic association was made when *RNASEL* was identified as the hereditary prostate cancer 1 (HPC1) locus on chromosome 1q25 [11,12,13,14,15]. Novel roles and mechanisms have continued to emerge, such as the correlation between a 37 kDa truncated form of RNase-L and chronic fatigue syndrome [16,17,18]. Other non-canonical functions for RNase-L include the induction of senescence and shortened lifespan, cellular differentiation, colitis susceptibility, the demyelination of axons, lipid storage, and the development of diabetes [19,20,21,22,23,24]. Although quite diverse, the mechanisms behind this regulation likely rely on transcriptional and post-transcriptional regulation of cellular mRNAs by RNase-L (reviewed in depth by Brennan-Laun *et al*.) [25]. More closely in line with its antiviral beginnings, the infection-associated role of RNase-L has been expanded in breadth to include antibacterial activity, immune cell regulation, the induction of IFN-β and autophagy, and most recently, the maintenance of cytoskeletal integrity and barrier function [26,27,28,29,30,31,32]. This association with the cytoskeleton places RNase-L amongst a growing group of innate immune proteins that are regulated by, or interact with, the cellular architecture. The significance of this novel mechanism of innate immune regulation will be discussed in detail later in this review.

## 2. Activation of the 2′-5′-Linked Oligoadenylates (2-5A)/RNase-L Pathway

**Figure 1:**
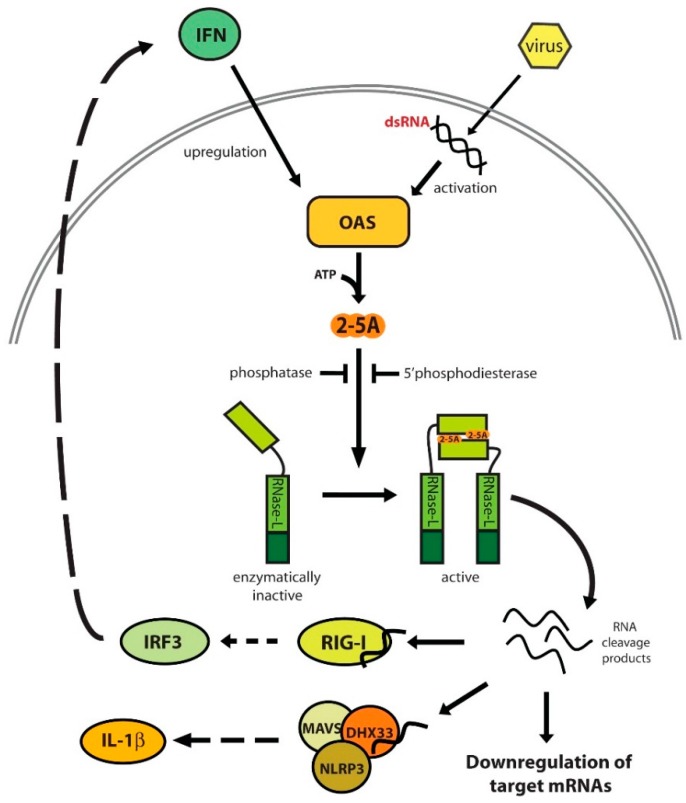
The 2′-5′-linked oligoadenylate (2-5A) System. IFN treatment induces the transcription of oligoadenylate synthetase (OAS), and virus infection produces dsRNA that activates OAS to synthesize 2-5A. Latent RNase-L binds 2-5A and oligomerizes into an active complex capable of cleaving ssRNA into retinoic acid-inducible gene-I (RIG-I) and nucleotide-binding oligomerization domain-like receptor 3 (NLRP3) inflammasome-activating small RNAs. Cleavage of target RNAs can regulate cellular and viral gene expression. RNase-L activation can be attenuated by phosphatase and 2′-phosphodiesterase mediated degradation of 2-5A. Solid arrows denote direct interactions and dashed arrows signify that intermediary steps occur.

The innate immune response is activated by the detection of pathogen-associated molecular patterns (PAMPs) or danger-associated molecular patterns (DAMPs) recognized by pattern recognition receptors (PRRs). These PRRs include retinoic acid-inducible gene-I (RIG-I)-like receptors (RLR) and DNA receptors that produce IFN, Toll-like receptors (TLR) that induce proinflammatory genes, and nucleotide-binding oligomerization domain-like (NOD) receptors (NLR) that activate the inflammasome. IFNβ is induced by both RLRs and certain TLRs and is a member of the type I interferons, a family of pleiotropic cytokines with antiviral, antibacterial, immunomodulatory, and antiproliferative activities [33]. Once expressed, IFNβ signals through its cognate receptor to induce the transcription of several hundred genes (IFN-stimulated genes, ISG) [34]. Amongst these ISGs is the family of OAS genes. There are four human OAS genes that encode 8–10 isoforms as a result of alternative splicing, and 12 murine OAS genes [35]. Each of these genes and their isoforms are differentially expressed in various tissues and can occupy different subcellular compartments, supporting the theory that they are non-redundant. In addition, not all OAS genes synthesize 2-5A capable of activating RNase-L. Activation-competent 2-5A must be in the form of a trimer or larger. OAS3 generates non-activating 2-5A dimers while OAS1 and OAS2 predominantly make activating trimers. OAS-like 1 does not produce 2-5A and recent studies show that OASL is induced following viral infection and binds to RIG-I to enhance the sensitivity of RIG-I activation upon binding viral RNA [36,37]. The OAS proteins are potently activated by dsRNA, which can occur in the form of viral genomes, replication intermediates, or transcripts from genes that overlap on opposite DNA strands (Figure 1). It can also be induced by single-stranded cellular RNAs, some of which have been identified [38,39]. This may be due to secondary structures in the transcripts that form the minimum 18–20 bp of dsRNA required for OAS activation [39,40]. Activation by cellular RNAs may explain how RNase-L functions in non-viral circumstances, such as senescence or tumorigenesis [41,42]. Following activation, OAS polymerizes ATP into 2-5A (p_x_5′A(2′p5′A)_n_; *n* ≥ 2) which can then bind to and activate RNase-L [3]. Activation of RNase-L is the only known function of 2-5A [6,10,43]. Once synthesized, 2-5A has a short half-life as it can be degraded within minutes by a 2′-phosphodiesterase (2′PDE) that breaks 2′-5′ bonds to remove 2-5A subunits from the activating multimer, or by phosphatases that remove the 5′ phosphate groups from 2-5A [44,45,46,47]. This likely contributes to the tight regulation of RNase-L activity required to prevent incidental or undesirably prolonged activation.

RNase-L is typically latent and expressed at low levels in most cell types. It possesses nine ankyrin repeats (the 9th being incomplete) in the N-terminus, and pseudokinase and nuclease domains in the C-terminus, which is also termed the kinase-extension nuclease (KEN) domain [4]. Nucleolytic activity is normally repressed due to interactions between the ankyrin repeat domain and the enzymatic domain, however upon binding to 2-5A, a conformational change in the KEN domain occurs to expose the pseudokinase and ribonuclease domains [43]. Recent crystal structure data indicate that 2-5A binds to the second and fourth ankyrin repeats and the pseudokinase domain. These interactions, in conjunction with binding between the pseudokinase domains of the two protomers, mediate dimerization and enzymatic activation within minutes [48,49,50]. The crystallization studies have also cast a new light on other canonical aspects of RNase-L activation. Previously, active RNase-L was generally believed to be a homodimer bound to 2-5A. *In vitro* activation analysis indicates that RNase-L can in fact oligomerize into complexes of two or more monomers and that at micromolar concentrations of RNase-L, 2-5A is dispensable for this oligomerization [49]. Whether physiologic concentrations of RNase-L reach these levels is unknown. Once active, RNase-L cleaves ssRNA, including cellular mRNA and rRNA as well as microbial RNAs [51,52]. The targeting of microbial RNAs has a significant impact on the ability of the virus or bacterium to replicate, as does the inhibition of cellular translation resulting from the cleavage of rRNA and ribosomal protein transcripts [6,26,41,53]. mRNA targeting can facilitate RNase-L-dependent regulation of gene expression through direct cleavage of transcripts or indirectly through the destabilization of transcriptional regulators [54]. Historically, this ribonuclease activity has been considered the major mediator of the biological activities of RNase-L.

## 3. Regulation of RNase-L and Its Nuclease Activity

In the event of pathogen infection, rapid activation of RNase-L increases the chance for clearance and host survival; however, uncontrolled activation of RNase-L could lead to cell death or dysregulated gene expression [13,15,26,55]. Therefore, RNase-L is tightly regulated by multiple mechanisms in order to maintain cell homeostasis. RNase-L is typically expressed at low levels in most mammalian cell types. It is marginally induced by IFNα/β and IFNγ, and sequence analysis primarily indicates tissue-specific and ubiquitous elements in the promoter [1,2,56]. Amongst these were sites for the specificity protein 1 (Sp1) transcription factor, which are present in many housekeeping genes. Recent findings show that overexpression of Sp1, which is associated with tumorigenesis, can induce RNase-L and many other RIG-I pathway members [57]. Although this may appear to contradict the role of RNase-L as a tumor suppressor, our lab has shown that RNase-L can have oncogenic activity in chronic myelogenous leukemia and this may contribute to that novel function in certain cell types [58]. In addition to transcriptional control, RNase-L mRNA is also regulated at the post-transcriptional level by the RNA-binding protein (RBP) human antigen R (HuR) [59]. HuR binds to AU-rich elements (ARE) typically found in the 3′UTRs of post-transcriptionally regulated mRNAs, and stabilizes them [60]. RNase-L has eight AREs, and deletion mapping indicates that there are both positive and negative regulatory elements in the 3′UTR. The two AREs closest to the 3′ terminus are stabilized by HuR and result in a several-fold increase in RNase-L expression [59]. Even low-to-moderate changes in RNase-L expression yield significant functional impact as RNA cleavage activity is disproportionately enhanced with even two-fold increases in expression [49]. Once the protein is made, RNase-L is susceptible to two forms of post-translational modification: ubiquitination and hydroxylation. Chase *et al.* [61] found that treatment of L929 cells with the phorbol ester phorbol-12-myristate-13 acetate (PMA) resulted in the rapid and almost complete reduction of RNase-L protein levels. This degradation was ablated by treatment with proteasome inhibitors. Similarly, ubiquitin modification of RNase-L was seen in the muscle tissue of a transgenic β5t mouse model that inhibited proteasomal chymotrypsin-like activity [62]. Hydroxylation of RNase-L was discovered in a screen for novel substrate interactors for the asparaginyl hydroxylase factor inhibiting hypoxia-inducible factor (HIF). Hydroxylation typically adds an –OH group to either Pro or Asn residues and has been shown to increase stability, inhibit signaling, or disrupt protein complex formations. The hydroxylation of RNase-L occurs at Asn-196 in the fifth ankyrin repeat domain; however, the effect of this modification is unknown [63].

RNase-L can also be regulated through several other protein–protein interactions. The best characterized RNase-L binding protein is the RNase-L inhibitor (RLI). RLI inhibits RNase-L as part of a complex containing RLI, RNase-L, and the translation termination-associated GTPase, eRF3 [64]. RLI, also known as ABCE1, is a member of the ATP binding cassette superfamily and, aside from its role as an RNase-L inhibitor, is believed to be involved in translation termination and ribosome recycling [65,66,67]. The mechanism for RNase-L inhibition is believed to be through direct binding with RLI, as RLI is not known to bind competitively with 2-5A. Regardless of the mechanism, however, overexpression or knockdown of RLI has inhibitory or promoting effects on the binding of RNase-L to 2-5A, rRNA cleavage, destabilization of mitochondrial mRNA, antiviral activity, and antitumor activity [68,69,70,71].

Three other interacting proteins are believed to possibly help target RNase-L to ssRNA substrates: the eukaryotic translation termination release factor eRF3, mitochondrial translation initiation factor (IF2mt), and tristetraprolin (TTP). eRF3 was discovered as an RNase-L interacting partner in 1993 but was originally termed RNABP (RNA binding protein) until its proper identification in 2005 by Le Roy *et al*. [72]. It binds to RNase-L as part of a complex and is believed to help facilitate its activity, since a presumed eRF3-inhibitory antibody, mAb3, blocks RNase-L-mediated rRNA cleavage and antiviral activity [73]. Although eRF3 does not bind 2-5A directly, the presence of 2-5A enhances the eRF3-RNase-L interaction, again suggesting that eRF3 is important to RNase-L function. eRF3, in conjunction with eRF1, mediates the release of the polypeptide chain from the ribosome during translation termination. It also interacts with RLI and the poly(A)-binding protein (PABP), which facilitates ribosome recycling by reinitiating translation after termination [74]. RNase-L localization with this translation termination complex places it in close proximity to potential mRNA targets as they are translated on polysomes. Actively translating RNAs adopt a closed-loop conformation in which the 3′UTR forms an exposed loop that may be accessible to regulatory factors. When active, RNase-L can outcompete PABP for eRF3 interaction in the termination complex, displacing it and providing RNase-L with access to mRNA substrates [25,72]. One caveat to this targeting model is that while ribosomes translate all mRNAs, RNase-L is selective, indicating that other factors are needed for specific targeting.

Similar to the accessibility that eRF3 may provide RNase-L to its targets, another binding partner may serve the same function in the mitochondrion. Although RNase-L is generally considered to be cytosolic, it is also found in the nucleus and mitochondria, where it has been shown to downregulate the expression of several mitochondrial RNAs (mtRNA), such as cytochrome b, ATPase6, and cytochrome oxidase subunit II [68,75,76]. In support of a role for RNase-L regulation of mitochondrial RNAs is the observation that the RNase-L regulators OAS, RLI, and 2′PDE have also been observed in mitochondria, and their modulation impacts the expression of RNase-L mitochondrial targets correspondingly [68,77,78]. In a yeast-two hybrid screen using RNase-L as bait, mitochondrial translation initiation factor (IF2mt) was isolated as a potential interactor and confirmed in rabbit reticulocyte lysate. IF2mt is a nuclear-encoded translation factor that delivers N-formyl methionyl-tRNA to the P-site of the mitochondrial ribosome during initiation. Using a human T cell lymphoma cell system in which IFNα induces RNase-L-dependent mtRNA degradation and cell death, it was shown that RNase-L activity required active translation. In addition, if excess IF2mt was introduced to the system so that RNase-L was competed away from translation initiation complex-bound IF2mt, the degradation of mtRNAs was suppressed, resulting in an increase in proliferation and a decrease in apoptotic signaling [76]. These data indicate that proximity to ribosome-bound mtRNAs is necessary for their RNase-L-dependent degradation. Therefore, like eRF3, IF2mt may facilitate accessibility, while other mechanisms must exist to provide specificity.

A third interacting protein may aid in providing this RNase-L target specificity. Our lab has shown that TTP can bind to RNase-L and coordinately regulate serum response factor (SRF) mRNA destabilization [54]. TTP is an ARE-binding protein that controls inflammation and suppresses tumorigenicity through the decay of target mRNAs. It binds these mRNAs using two zinc finger domains, often recognizing a UUAUUUAUU nonamer sequence, but utilizes other enzymes to mediate degradation [79,80]. Our data indicate that TTP may guide RNase-L to specific targets like SRF mRNA, facilitating RNA cleavage by the nuclease, possibly at the UU and UA dinucleotide sites within the TTP nonamer. TTP and RNase-L share a subset of targeted RNAs, including IL-8, HMGA2, and TTP itself, indicating that this mechanism may not be unique to SRF [54]. Also lending support to this cooperative function is the identification of the nuclease Regnase-1. Regnase-1 contains both endoribonucleolytic activity like RNase-L, and a CCCH-type zinc finger that mediates mRNA binding similar to TTP [81,82]. It essentially combines the activities of RNase-L and TTP into a single enzyme, setting precedent for this cooperative function. Many RNase-L substrates are not shared with TTP, indicating that additional mechanisms are likely involved and other ARE-binding proteins (AREBP) may serve as potential targeting candidates. AREBP post-transcriptional regulators are strong contenders because although no definitive consensus recognition site exists for RNase-L, its preference for UU and UA dinucleotides is identical to those found in AREs.

## 4. The Antimicrobial Actions of RNase-L

### 4.1. RNase-L Antiviral Activity

RNase-L was initially discovered and characterized as a mediator of type I IFN antiviral activity. Although cleavage of RNA virus genomes appeared as the most direct mechanism of action, other important pathways have become evident, such as the regulation of host gene expression, stimulation of IFNβ production, activation of the NACHT, LRR, and PYD-containing protein-3 (NLRP3) inflammasome, and maintenance of the cell’s structural barrier to infection [27,55,83,84]. These will be discussed in further detail below. Here we highlight some of the decades of work demonstrating the importance of RNase-L to the replication and survival of specific viruses; a full examination of this subject is provided by R.H. Silverman [85]. RNase-L has been shown to play a role in antiviral activity against both RNA viruses (*Picornaviridae*, *Reoviridae*, *Togaviridae*, *Paramyxoviridae*, *Orthomyxoviridae*, *Flaviviridae*, *Coronaviridae* and *Retroviridae*) and DNA viruses (*Poxviridae*, *Herpesviridae*, and *Polyomaviridae*). The prototypical virus for demonstrating RNase-L activity is the picornavirus, encephalomyocarditis virus (EMCV). OAS has been isolated while bound to both the positive and negative strands of the EMCV genome, indicating that it can be activated by the double-stranded replicative intermediate of the positive-stranded RNA genome [86,87]. Following 2-5A production, activated RNase-L then cleaves both cellular and viral RNAs, which not only directly inhibits virus replication, but produces small RNAs capable of stimulating RIG-I activation and subsequent downstream IFNβ transcription to protect surrounding tissue by creating an antiviral state [27,53]. Overexpression of RNase-L has been shown to suppress EMCV replication whereas dominant-negative RNase-L inhibits IFN-induced protection against infection, indicating the effectiveness of RNase-L antiviral activity [14,53]. In order to determine whether these effects were physiologic *in vivo*, RNase-L knock-out (KO) and RNase-L wild type (WT) mice were infected with EMCV, and wild type mice exhibited both a better rate of survival and delayed onset of death as compared to knockout mice [15]. Though EMCV is sensitive to RNase-L activity, other members of the *Picornaviridae* family, such as Theiler’s virus and poliovirus, have adapted mechanisms for evading RNase-L activity [88,89,90,91].

The evolution of these and other inhibitors are a testament to the antiviral potency of the OAS/RNase-L pathway. In an *in vitro* hepatitis C virus (HCV) infection model, RNase-L was demonstrated to cleave the genome at UU and UA dinucleotides into 200–500 bp fragments [92]. Sequence analysis indicated that UU and UA dinucleotides are underrepresented in the HCV genome and that IFN-sensitive HCV genotypes contain more UU and UA dinucleotides than IFN-resistant genotypes [93]. This minimizing of potential RNase-L cleavage sites could be an effective evasion strategy by this chronic pathogen [94]. Influenza A virus (IAV) has also been shown to be cleaved at discrete locations in both the positive and negative RNA strands of the replicative intermediate genome, but only in the absence of the IFN inhibitor NS1 [95,96]. NS1 binds to and sequesters dsRNA in order to prevent the activation of the viral sensors RIG-I, OAS, and the IFN-induced protein kinase PKR [97]. Since this mechanism not only inhibits the OAS/RNase-L pathway, but other potent antiviral actions including the induction of IFNβ, other viruses such as vaccinia virus, human immunodeficiency virus (HIV), and reovirus, have also adopted this strategy [85]. This sequestration of activating RNAs may not be completely efficient throughout the course of infection, potentially resulting in low levels of free 2-5A. Therefore, HIV also induces the expression of RLI to prevent the binding of any free 2-5A to RNase-L, further safeguarding against activation [70,98]. A relatively new mechanism for inhibiting RNase-L activity was identified in the coronavirus mouse hepatitis virus (MHV). The MHV *ns2* gene encodes its own 2′phosphodiesterase to degrade 2-5A. When compared to wild type virus, the mutant virus ns2-H126R that expresses catalytically inactive ns2, was unable to inactivate 2-5A and inhibit RNase-L activation, thus the development of hepatitis and replication in the liver was unimpaired. This demonstrates that MHV-ns2 is a potent antagonist of the OAS/RNase-L pathway [99]. A more detailed analysis of these and other viral evasion mechanisms are reviewed by Drappier *et al.* [100], highlighting the importance of RNase-L as an antiviral effector.

### 4.2. RNase-L-Mediated Antibacterial Activity

Although the type I IFNs were originally discovered as agents of antiviral activity, their role in defense against other pathogens has been a continually expanding field, particularly since the discovery of the TLRs. In 2008, our lab provided the first evidence of antibacterial activity by RNase-L. We demonstrated that RNase-L KO mice are more susceptible to sublethal infection by both *Escherichia coli* (*E. coli*) and *Bacillus anthracis* (BA) than WT mice. Multiple mechanisms were found to contribute to this sensitivity, including changes in gene regulation that likely contributed to immune cell dysfunction. Specifically, IFNβ, the proinflammatory cytokines tumor necrosis factor-α (TNFα) and interleukin-1β (IL-1β), and a protease involved in endolysosomal maturation, cathepsin E, were all dysregulated in RNase-L KO mice and purified peritoneal macrophages. This likely contributed to the decreased bactericidal activity observed in infected macrophages, altered macrophage maturation, and differences in neutrophil and lymphocyte recruitment. These changes in proinflammatory gene expression were also seen in response to dsRNA, lipopolysaccharide, and CpG DNA stimulation of TLRs 3, 4, and 9, respectively [26]. On the heels of this work, it was discovered that *E. coli* RNA could activate RNase-L induction of IFNβ and that RNase-L also plays an important part in gut immunity [23]. Therefore, our lab investigated the role of RNase-L in the pathogenesis of the intestinal pathogen enteropathogenic *E. coli* (EPEC). In a novel antimicrobial role for RNase-L, we found that it helps maintain the intercellular barrier formed by intestinal epithelial cell (IEC) monolayers, preventing pathogen translocation from the gut into the host. Similar to our previous studies, we also saw dysregulation of IFNβ and TNFα as well as novel regulation of the tight junction proteins occludin and claudin-1, which undoubtedly contributed to the defects in barrier function [84]. Many types of bacteria may activate RNase-L through endocytosis and either escape of the bacterium or leakage of RNA into the cytosol during infection. However, since RNA in actively replicating bacteria is not exposed to cytosolic RNase-L, it is likely resistant to direct cleavage as an effector mechanism. Therefore, as touched on above, many of the other antimicrobial functions of RNase-L may be more prominent in these types of infections.

### 4.3. Alternative Cellular and Innate Immune Activities

As exemplified by the examples of antibacterial activity, RNase-L utilizes a variety of mechanisms to combat microbial infections. Early investigations into antiviral activity focused on direct cause-and-effect events such as RNase-L activation and virus genome cleavage to inhibit replication. The expansion of studies into DNA viruses and then bacteria have corresponded with broadening ideas in the field about RNase-L functions via more indirect antimicrobial activities, including apoptosis, immune modulation, gene regulation, autophagy, and structural integrity. One of the first alternative mechanisms was elucidated by Castelli *et al.* [12,13], who demonstrated that RNase-L mediated apoptosis in response to activation by virus infection or dsRNA. Zhou *et al.* [15] confirmed this role for RNase-L in Fas, TNFα, and α-CD3 antibody-induced apoptosis. Further characterization showed that RNase-L-dependent cell death resulted in the release of cytochrome c from the mitochondria and required caspase-3 activation [101]. This early described role for RNase-L may be partially coordinated with a newly discovered function in the induction of autophagy. Autophagy is a stress response that induces the recycling of proteins, damaged organelles, and pathogens by isolating them within a double-membraned autophagosome that then fuses with a lysosome in order to degrade its contents into reusable components [102]. RNase-L induces autophagy in response to viral infection, or more directly by 2-5A. The signaling involved in activating this process involves both c-Jun N-terminal kinase and PKR, resulting in the degradation of p62 (SQSTM1), LCSBI/LCS3BII conversion, and the accumulation of autophagosomes, all hallmarks of autophagy. RNase-L-induced autophagy was shown to be detrimental to virus replication in the early stages of infection and then advantageous in later stages, or under high infectious doses of virus [29,103]. It has previously been shown that autophagy suppresses apoptosis at early timepoints in order to give the cell time to recover. If the activating stressor continues or autophagy is excessive, the cell can then switch in favor of a cell death response [104]. Recently, Siddiqui *et al.* [30], have shown that RNase-L cleavage products can promote this switch to apoptosis by activating caspase-3 to cleave Beclin-1, which then translocates to the mitochondria to induce cytochrome c release.

Like the induction of autophagy, the exact mechanisms for many of the cellular functions of RNase-L are unknown. It is broadly believed that the regulation of gene expression plays a major role in facilitating these activities, such as the modulation of the immune response. The gene most notably regulated by RNase-L is IFNβ. In 2007, Malathi *et al.* [27] showed that activated RNase-L cleaved cellular and viral RNAs into small RNAs (<200 bp) that often form a duplex. These small RNAs are capable of stimulating RIG-I and MDA5 (melanoma differentiation associated gene-5) to activate mitochondrial antiviral signaling protein (MAVS) and induce the subsequent translocation of interferon regulatory factor 3 (IRF3) to the nucleus to drive transcription of IFNβ. Once secreted, IFNβ can not only create an antiviral state in surrounding cells, but it can affect immunoglobulin class switching in B cells as well as T and NK cell activation, affecting the adaptive immune response to pathogens [105,106,107]. Skin allograft rejection and contact hypersensitivity experiments conducted in RNase-L KO and WT mice revealed a five day delay in allograft rejection in the KO mice and a reduction of inflammatory infiltrates [31]. This may indicate a defect in T-cell priming or immune cell trafficking; however, further studies are required to determine the exact mechanism for RNase-L activity. The possible involvement of RNase-L in T cell function is not surprising, as RNase-L KO mice have enlarged thymuses, likely due to defects in apoptosis. Other investigations of immune cell functions have shown that, in addition to our work with *E. coli* and BA infections above, macrophages deficient in RNase-L exhibit decreased migration, endocytic activity, and proinflammatory gene regulation [26,32]. These data clearly indicate that RNase-L has an important role in immune cell function, including the adaptive response.

In addition to type I IFN and TLR signaling, which were shown to be regulated by RNase-L during bacterial infection, another family of innate immune regulators also interacts with the OAS/RNase-L pathway. Nucleotide-binding and oligomerization domain (NOD)-like receptors (NLR) detect microbial products and are divided into two subsets, those that drive mitogen-activated protein kinase (MAPK) and nuclear factor-κB (NF-κB) signaling (such as NOD1 and NOD2), and those that activate the inflammasome, leading to caspase-1, IL-18, and IL-1β processing and secretion (including NLRPs 1, 3, and 4 and AIM2) [108]. Recently, RNase-L has been shown to play a role in the activation of the NLRP3 inflammasome. In this process, RNase-L is activated following virus infection to generate small RNA cleavage products that bind to the DExD/H helicase DHX33. This leads to the formation of a DHX33, NLRP3, and MAVS complex and increased IL-1β production [83]. Association with the other subset of NLRs occurs when OAS binds to NOD2. NOD2 is activated by the peptidoglycan breakdown product muramyl dipeptide as well as viral ssRNA. Overexpression of NOD2 when OAS is active leads to increased RNase-L activity, suggesting a connection between the pathways [109]. Any effects of OAS interaction on NOD2 activation of MAPK or NF-κB signaling are unknown.

## 5. RNase-L and the Cytoskeleton

The long-standing model of RNase-L activity involves low levels of expression in the cytosol in an inactive conformation, activation by 2-5A, and cleavage of ssRNA in order to exert its biological functions. Though all of this is well-documented, it does not address whether constitutively expressed RNase-L serves a physiologic role in the absence of 2-5A. In addition, while RNase-L is widely considered to be a cytosolic protein, it is also associated with the nucleus, mitochondria, and cytoskeleton [76,110,111,112]. Although RNAs are accessible in all of these cellular compartments, it prompts the question whether there are alternative functions for this localization. Early insights into this were provided by the 1998 study by Tnani *et al.* [112], in which they used biochemical methods to detect RNase-L in the cytoskeletal fraction of cell extracts. They demonstrated that RNase-L associated with the cytoskeleton in a conformation that did not allow it to bind 2-5A, which supports a model in which cytoskeletal association is incompatible with RNase-L activity. Indeed, it is not surprising that the dramatic structural changes that occur upon RNase-L activation would impact its interactions with cytoskeletal components. Treatment with PMA, a known inducer of cytoskeletal alterations, dissociated RNase-L from the cytoskeleton and corresponded with its phosphorylation by protein kinase C (PKC) [112,113,114]. Thus, changes in cell structure may induce the phosphorylation of RNase-L as a mechanism to promote its release from the cytoskeleton or induce conformational changes that enable activation [61,112]. This would suggest a model in which RNase-L bound to the cytoskeleton is inactive, however, PMA or the subsequent actin reorganization may disrupt this association so that it can bind 2-5A and initiate downstream signaling and effector functions. More recently, Gupta *et al.* [111] performed a proteomic analysis of RNase-L binding proteins and found that over 50% of those identified were involved in cytoskeletal and motor assembly. These included β-actin, troponin I, myosin heavy chain 9, fibronectin precursor and an uncharacterized protein with homology to myosin binding protein C, slow type isoform CRA_j. In addition to this screen, three additional interacting proteins have been more fully characterized and contribute towards a new model of cytoskeleton-associated RNase-L activity.

The first of these interacting partners is a scaffold protein involved in actin reorganization, identified as the isoleucine-glutamine (IQ) motif-containing Ras GTPase-activating-like protein 1, or IQGAP1. IQGAP1 is a 190 kDa, ubiquitously expressed protein that shares a number of biological functions with RNase-L, including the regulation of migration, proliferation, and differentiation. In addition, both proteins regulate the formation of tight junctions (TJ). IQGAP1 alters TJ assembly by reducing the recruitment of claudin 2 and increasing the assembly of claudin 4 into TJ complexes. It also diminishes barrier function by inhibiting the Cdc42-JNK signaling pathway [115]. Conversely, RNase-L promotes the expression of TJ proteins claudin 1 and occludin, enhances barrier function, and prevents the transepithelial migration of pathogens such as EPEC [84]. IQGAP1 functions as a multifunctional scaffolding protein, involving interactions with over 50 binding partners thus far [116,117]. One of the main roles for IQGAP1 is in actin polymerization. It can help maintain Cdc42 and Rac1 in their active, GTP-bound state by inhibiting GTP hydrolysis. In the case of Cdc42, this activity keeps WASP (Wiscott-Aldrich syndrome protein) bound to Cdc42, stabilizing it and maintaining its interactions with actin-related protein 2 and 3 (Arp2/3) and globular actin. This preservation of the interactions enhances actin polymerization and branching [118]. IQGAP1 was originally identified as an RNase-L interacting protein in screens searching for a mechanism for RNase-L-mediated cell death induced by the drug 1-(3-*C*-thynyl-β-d-*ribo*-pentofuanosyl) cytosine (ECyd). ECyd is a cytotoxic nucleoside analogue that is active against cancer cells, and both RNase-L and IQGAP1 were necessary for ECyd-induced apoptosis in human fibrosarcoma HT1080 cells. ECyd treatment enhanced IQGAP1 interaction with RNase-L, however, no significant function for this interaction was determined [119,120].

We recently identified filamin A (FLNA), an actin-binding protein that functions as a scaffold linking many diverse proteins to the cytoskeleton, as a second RNase-L interacting protein. This interaction revealed a novel biological function for RNase-L in membrane integrity and was abrogated upon RNase-L activation by either 2-5A or EMCV infection. Complex formation did not require RNase-L nuclease or dimerization functions, but did require the N-terminal ankyrin repeat domains. Other RNase-L interacting proteins have, somewhat surprisingly, bound to the C-terminus, possibly indicating that RNase-L may bind multiple proteins simultaneously. In addition, binding to FLNA did not inhibit or enhance the activation or nucleolytic activities of RNase-L. Examination of the potential reciprocal consequences of the interaction on actin dynamics found that RNase-L KO primary murine embryonic fibroblasts (MEFs) had a 30% reduction in actin filaments compared to WT MEFs. Further probing into this role for RNase-L in actin cytoskeleton function demonstrated that RNase-L inhibited the endocytosis of both an inert synthetic agent and viral particles. Significantly, inhibition of virus entry was independent of endoribonuclease activity, providing the first evidence of a non-catalytic mechanism of RNase-L action. All other functions for RNase-L have been shown, or presumed to be the result of RNA cleavage leading to altered regulation of gene expression via direct or indirect degradation mechanisms [25]. The diminished uptake of virus in RNase-L-expressing cells was sufficient to inhibit overall virus production and this was complemented by similar results in the presence or absence of FLNA [28]. These data provide a new model for RNase-L activity as a structural component of the cytoskeletal barrier that refracts the actin reorganization involved in the endocytosis of viral particles. This localization may also place RNase-L in immediate proximity to virus that is able to bypass this first layer of host defense in order to hasten the antiviral response via its canonical nuclease-dependent functions. Activation of RNase-L may then cause a conformational change that induces its release from FLNA. Although FLNA does not alter the capacity of virus to activate RNase-L, the interaction may aid in preventing incidental oligomerization of latent RNase-L monomer, adding to the many layers of regulation needed to keep RNase-L activity in check. These findings were the first to truly characterize a function for RNase-L in the cytoskeleton.

**Figure 2:**
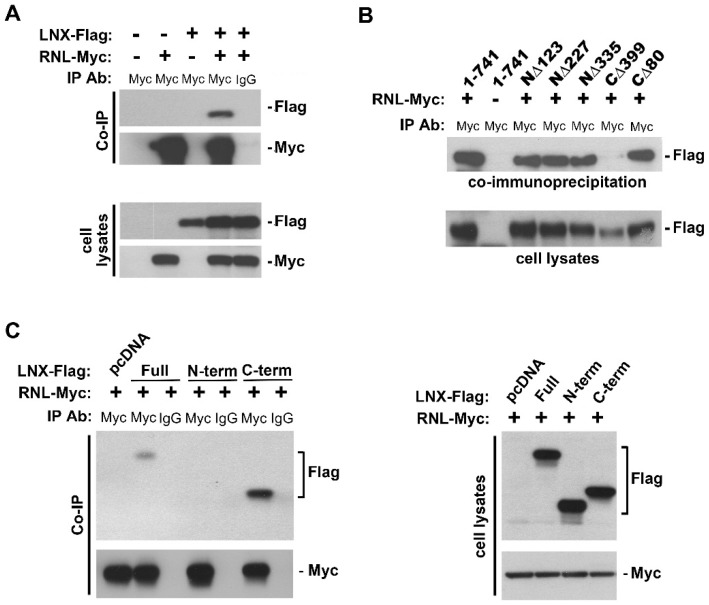
RNase-L interacts with LNX. (**A**) 293T cells were transfected with pCMV3-RNL-myc, pcDNA3-hLNX-Flag, or the respective empty vectors using Lipofectamine 2000 (Invitrogen). Cell lysates were immunoprecipitated (IP) using anti-Myc-tag or IgG control antibodies bound to protein A/G agarose beads. Bound protein complexes were detected by Western blot analysis using the antibodies as indicated; (**B**) RNase-L and (**C**) LNX deletions were used in IP studies as in (**A**) to determine their respective interaction domains [43,127]. RNase-L deletions were subcloned from the pGEX4T3 plasmid (kindly provided by R.H. Silverman) into the eukaryotic expression plasmid pCMV3B using the BamHI and XhoI restriction sites.

Our lab has also identified a third interacting protein that links RNase-L to the cytoskeleton, the ligand of numb protein X-1 (LNX). LNX is a cytoskeleton/TJ-associated protein that was isolated through a yeast two-hybrid screen using RNase-L as bait. LNX is an E3 ubiquitin ligase that contains a RING finger in its *N*-terminus, which mediates ubiquitin ligase activity, and four PDZ domains (named after the proteins postsynaptic density 95, Drosophila disc large tumor suppressor, and zonula occludens-1) that facilitate protein–protein interactions and are present in many TJ/cytoskeletal proteins [121,122]. The interaction between LNX and RNase-L was confirmed in 293T cells and mapped to the C-terminus of RNase-L and the PDZ domain-containing C-terminus of LNX (Figure 2). LNX also functions as a scaffold protein. In addition to several characterized interactions, a proteomic screen for potential LNX ubiquitylation substrates identified 64 candidate interactors, and yeast data suggest that the LNXp80 and p70 isoforms can form homo- or hetero-oligomers, potentially forming large complexes [123]. Together, these E3 ligase and scaffolding functions are thought to mediate the physiologic activities of LNX, including cell signaling, cell cycle progression, tight junction reorganization, epithelial-to-mesenchymal transition, and its association with glioblastoma [124,125,126,127,128,129].

**Figure 3:**
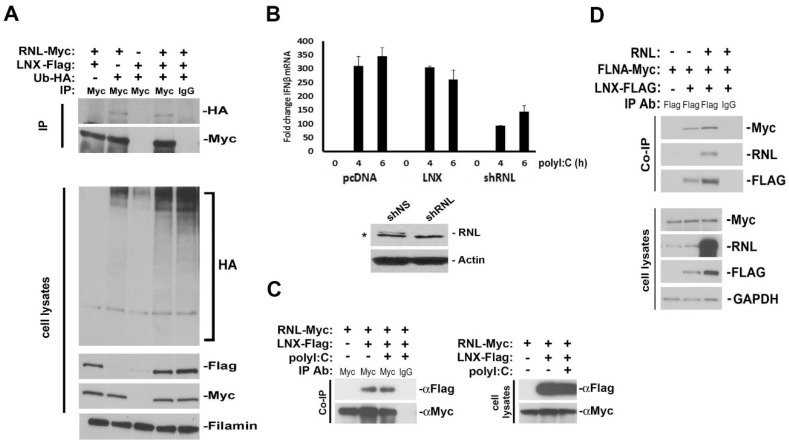
Characterization and consequences of LNX expression. (**A**) Ubiquitylation of RNase-L is not enhanced by LNX expression. 293T cells were transfected with LNX, RNase-L, and HA-tagged ubiquitin (Ub). The presence of Ub-HA-conjugated RNase-L was determined by Western blot; (**B**) Hela cells were transfected with the indicated vectors. After 48 h, cells were transfected with polyI:C for 4 and 6 h and then analyzed by RT-qPCR for IFNβ expression, relative to rpl13a mRNA. Error bars represent standard deviation of two experiments. Western blot demonstrates shRNA knockdown of RNase-L expression. * indicates a non-specific band (**C**) 293T cells were transfected with LNX and RNase-L. After 48 h, cells were transfected with polyI:C for 6 h and then interaction was analyzed by IP; (**D**) Analysis of FLNA-myc and LNX-Flag interaction. FLNA and LNX were transfected into 293T cells in the presence or absence of RNase-L. Cell lysates were immunoprecipitated for LNX-Flag and Western blots probed for FLNA interaction. The presence of exogenous RNase-L did not alter the interaction.

Interaction with LNX validated our model for a role for RNase-L in the cytoskeleton. Although a logical function for this relationship, LNX does not ubiquitylate RNase-L, a known target of modification, and like filamin A (FLNA), it also does not alter IFNβ induction by RNase-L cleavage products in response to activating conditions (Figure 3A,B). Contrary to the relationship with FLNA, however, LNX interaction with RNase-L is not abrogated under activating conditions (Figure 3C). This lack of regulation or modification may indicate that LNX is functioning as a scaffold when binding to RNase-L or that RNase-L is regulating LNX activity. Like RNase-L and IQGAP1, LNX regulates multiple tight junction (TJ) proteins [126,130,131,132]. Claudin-1 is ubiquitylated by LNX, leading to its lysosomal degradation and a reduction in TJ strands. It also colocalizes with claudin-2 in late endosomes and lysosomes, suggesting that it may mediate the endocytosis of claudins to lysosomes for degradation [126]. Given our previous findings that RNase-L upregulates claudin-1 and occludin following enteropathogenic *E. coli* (EPEC) infection, it is plausible that RNase-L may normally bind to LNX and prevent its ubiquitylation and degradation of claudin-1. Whether this is sufficient to maintain the barrier function observed in RNase-L-expressing cells compared to deficient cells, or if other factors including FLNA and IQGAP1 also contribute, has yet to be determined. In support of a cooperative complex however, is data indicating that FLNA and LNX are capable of interacting with each other in a manner that is not altered by RNase-L overexpression (Figure 3D). Given that FLNA binds to the *N*-terminus of RNase-L and LNX binds to the C-terminus (Figure 2B), it is plausible that RNase-L could bind to both proteins simultaneously in a complex at the interface of TJs and the actin cytoskeleton.

**Figure 4:**
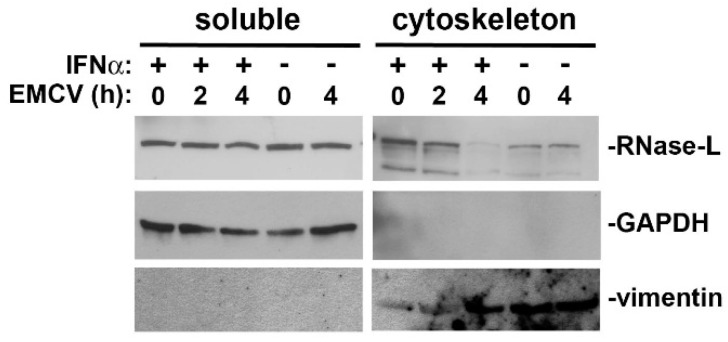
Analysis of RNase-L association with the cytoskeleton. Caco-2 cells were treated with 500 U/mL hIFNα for 24 h and then infected with encephalomyocarditis virus (EMCV; MOI = 10) for 2 and 4 h. Soluble and cytoskeletal protein fractions were collected using the ProteoExtract Cytoskeleton Enrichment and Isolation Kit (Millipore) according to the manufacturer’s protocol. GAPDH and vimentin were used to evaluate soluble and cytoskeleton fraction purity, respectively.

**Figure 5:**
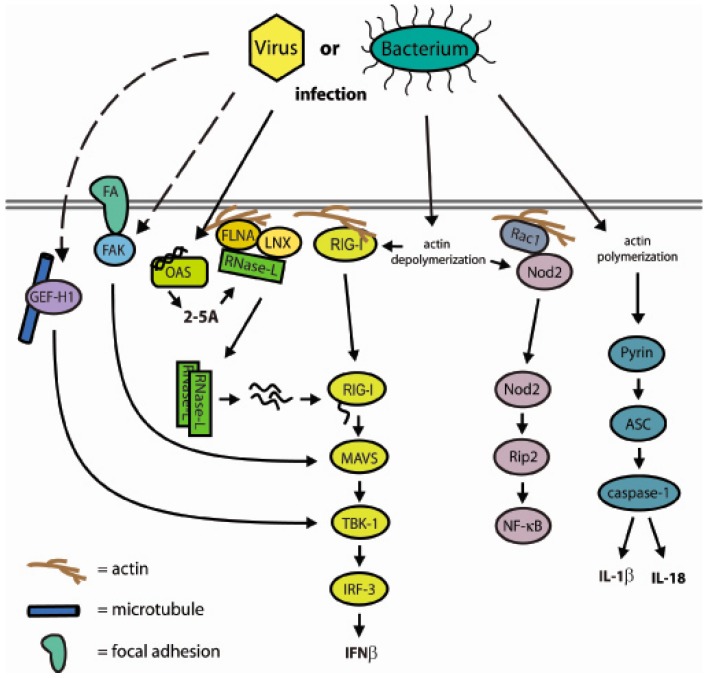
Model of cytoskeletal interactions with components of innate immunity. Microbial infection creates perturbations in cytoskeletal interactions or actin polymerization that function as sensors to activate the innate immune response. The IFNβ and inflammasome pathways are key targets. Solid arrows denote direct interactions and dashed arrows signify that intermediary steps occur.

Collectively, the data suggests a model in which FLNA, LNX, and RNase-L form a complex associated with the actin cytoskeleton. During resting conditions, this complex supports membrane integrity to inhibit endocytosis or viral entry into the cell. If the virus breaches the membrane barrier and activates OAS to produce 2-5A, then the RNase-L-FLNA interaction is abolished and RNase-L is released from the cytoskeleton ([23] and Figure 4). Since the N-terminus of RNase-L is bound and then released by FLNA and the C-terminus of RNase-L is still capable of binding to LNX, it is likely that 2-5A binding to RNase-L causes a conformational change to LNX-bound RNase-L that induces the dissociation of FLNA from LNX. This may not only release RNase-L from FLNA but also alter LNX localization. RNase-L is then free to oligomerize into an active endoribonuclease, cleave viral and cellular RNAs into RIG-I activators, and induce IFNβ expression to create a broader antiviral state (Figure 5).

## 6. Cytoskeletal Interactions as a Component of the Innate Immune Response

This model of RNase-L function parallels an emerging network of innate immune proteins that are directly associated with cytoskeletal components to serve roles both as a barrier to pathogen infection and to initiate signaling and effector functions upon infection. Although each protein has been identified and characterized singularly, this growing trend may indicate broader relationships between cytoskeletal functions and innate immune regulation and activity. Consistent with this view, bacteria and viruses both modulate actin polymerization as central components of infection and replication [133,134]. Furthermore, perturbation of the actin cytoskeleton activates cytoskeleton-associated PRRs [135,136,137]. These observations suggest that the cytoskeleton may serve as a sensor for infection and that pathogen-induced disruption of this structural matrix serves as a signal to activate an innate immune response. Here we describe specific innate immune mediators and their proposed roles in this model (summarized in Table 1). Understanding these interactions will likely reveal a novel and coordinated mechanism of cellular host defense.

*RIG-I*: Perhaps the first PRR discovered to be activated by the actin cytoskeleton was RIG-I. RIG-I was found to localize to the apico-lateral cell junctions and tight junctions of polarized intestinal epithelial cells as well as actin-rich membrane ruffles in non-polarized cells. Physiologically, RIG-I expression positively correlated with cell migration, which requires both actin polymerization and depolymerization, indicating that RIG-I may contribute to cytoskeletal function as well [137]. In fact, LPS-activated, RIG-I-deficient macrophages have impaired phagocytosis and actin polymerization [138]. When cells were treated with the actin depolymerizing drug cytocholasin-D (cytD), RIG-I relocalized to punctate domains and induced both IFNβ and NF-κB signaling [137]. Although the use of cytD may mimic the actin depolymerization that can occur during virus infection, cytD activation of RIG-I and induction of IFNβ expression in the absence of known viral agonists supports the role of the cytoskeleton as an innate immune sensor.

**Table 1:** Cytoskeletal interactions with members of the innate immune response.

Innate Immune Protein	Cytoskeleton Protein	Characterization of Interaction	Reference
*Cytoskeleton regulated activation*			
NOD2	Rac1	Recruits NOD2 to membrane ruffles Actin depolymerization leads to activation and release to insoluble cell fraction	Legrand-Poels *et al.* [136]
RIG-I	Unknown	Localizes to tight junctions and membrane ruffles Actin depolymerization leads to relocalization to punctate domains and IFNβ induction	Mukherjee *et al.* [137]
MAVS	FAK	Virus infection induces FAK relocalization from focal adhesions to binding MAVS at the mitochondrion, inducing FAK-dependent IFNβ expression	Bozym *et al.* [139]
TBK1	GEF-H1	RLR activation induces GEF-H1 dephosphorylation, release from microtubules, and binding to TBK1	Chiang *et al.* [140]
Pyrin	Unknown	Actin polymerization induced by mutation of the Wdr1 depolymerizing factor activates the pyrin inflammasome to produce elevated IL-18	Kim *et al.* [135]
Caspase-1	Flightless	Inhibits caspase-1 activation and subsequent IL-1β maturation	Li *et al.* [141]
*Innate regulation of the cytoskeleton*			
ASC	Dock2	ASC stabilizes Dock2 mRNA which mediates Rac-dependent actin polymerization	Ippagunta *et al.* [142]
RNase-L	Filamin A	RNase-L KO MEFs express decreased levels of polymerized actin RNase-L monomer binds to Filamin A to inhibit virus entry	Malathi *et al.* [28]
PKR	Gelsolin	Latent PKR inhibits gelsolin from severing actin filaments	Irving *et al.* [143]
Caspase-11	Aip1	Caspase-11 enhances Aip1-dependent actin polymerization	Li *et al.* [144]
ADAP2	Arf6	Induces membrane ruffling and macropinocytosis Associates with and controls actin dynamics Inhibits Dengue virus and VSV	Shu *et al.* [145]
*Undetermined function of the interaction*			
IFITM1	Occludin, Claudin1, and ZO-1	Enhances the interaction between HCV receptors occludin and CD81, inhibiting virus entry	Wilkins *et al.* [146]
RNase-L	IQGAP1	Unknown	Sato *et al.* [120]
	LNX1	Unknown	Figure 2 and Figure 3

*MAVS*: A second mechanism of actin-induced IFNβ involves the interaction of MAVS (mitochondrial antiviral signaling protein) and focal adhesion kinase (FAK). MAVS is an adaptor protein that binds to RIG-I or MDA-5 after they interact with their corresponding RNA activators. This interaction then leads to the activation of the kinases TANK binding kinase-1 (TBK-1) and inhibitor of κB kinase-ε (IKKε), which phosphorylate IRF3 and induce IFNβ expression. As its name denotes, FAK is a protein tyrosine kinase localized to focal adhesion (FA) contacts with the extracellular matrix (ECM). The FAs relay signals from the ECM to the cytoplasm and are therefore extremely sensitive to activation by alterations in the actin cytoskeleton. As a result, virus infection was shown to relocate FAK from FAs to the mitochondrion where it binds to MAVS to induce IFNβ. FAK-deficient cells are defective in IFNβ expression and more susceptible to virus infections, indicating that FAK may serve as an important link between virus-induced cytoskeletal disruption and IRF3 signaling [139].

*TBK1*: GEF-H1 is a RhoA guanine nucleotide exchange factor that binds to the dynein motor complex on microtubules. Past work demonstrated that it activates NOD1, NOD2, and phosphorylates Rip2 in response to bacterial infection [147,148]. Recently, it has also been shown to be critical for IFNβ expression in response to nucleic acid PAMPs. Upon activation of RLRs, GEF-H1 is dephosphorylated and released from microtubules, freeing it to bind to TBK1 and thus activating IRF3 to express IFNβ. This interaction between GEF-H1 and TBK1 is dependent on functional microtubules. For undetermined reasons, this regulation is only seen in response to RIG-I, MDA-5, and STING, but not TLR activation [140].

*NOD2:* NOD2 can be activated by bacterial PAMPs as well as viral ssRNA to induce NF-κB and IRF3 signaling [108]. Legrand-Poels *et al.* [136], have shown that NOD2 binds to Rac1, a GTP-binding Rho family member that regulates actin polymerization, and recruits it to actin-rich membrane ruffles. There it is sequestered in a latent state until actin disruption, potentially by infection, releases it to begin signaling. Treatment of cells with cytD or the actin polymerization inhibitor latrunculin B significantly increased NF-κB signaling and caused NOD2 to shift from the insoluble Triton-X-100 cell fraction to the soluble fraction. As previously described, OAS also binds to NOD2, which could potentially localize it to the cytoskeleton as well. This would place it in proximity to cytoskeleton-associated RNase-L for rapid, and perhaps localized, activation. In addition, the presence of NOD2 was shown to enhance RNase-L activation in response to dsRNA, possibly generating a more robust response to viral infection [109].

*ASC*: Inflammasome activation is induced by either PAMPS or DAMPS which typically stimulate the oligomerization of one of the NLRs, ASC (apoptosis-associated speck-like protein containing CARD; caspase recruitment domain), and caspase-1. This leads to caspase-1 activation, enabling it to cleave IL-1β and IL-18 into their mature forms [108]. ASC KO cells have been shown to be deficient in antigen uptake and presentation, independent of its inflammasome activity. Gene expression comparisons between WT and ASC KO cells identified a novel role for ASC in the stabilization of Dock2 mRNA. Dock2 is a guanine nucleotide exchange factor that mediates Rac-dependent actin polymerization; thus, in the absence of ASC, Dock2 levels are low, resulting in inactive Rac and deficient filamentous actin production [142]. This inability to polymerize actin would severely diminish active processes like endocytosis and cell migration. This ASC-mediated indirect regulation of actin polymerization may serve to stabilize the cytoskeleton and enhance barrier function.

*Pyrin*: Pyrin is an inflammasome receptor that interacts with microtubules and actin filaments. Utilizing a mouse model in which the actin depolymerizing factor Wdr1 was mutated, Kim *et al.* [135] demonstrated that the perturbation of actin resulted in autoinflammation that is dependent on the pyrin inflammasome and activation of IL-18. The Wdr1 mutation implies that in this system, excessive actin polymerization triggers this inflammasome activation; however, another group found that decreased polymerization resulting from bacterial toxins targeting Rho GTPases also activated the pyrin inflammasome [149]. This discrepancy may be a consequence of numerous differences in the two model systems, however both reports demonstrate a link between cytoskeletal and inflammasome components that requires further investigation.

*PKR*: PKR was found to interact with the actin-binding protein gelsolin, an enzyme that catalyzes actin cleavage and nucleation. Activation of PKR by dsRNA diminishes the interaction with gelsolin, as gelsolin is only capable of binding to PKR monomer and not the active dimeric form. In its latent state, PKR inhibits the ability of gelsolin to sever actin filaments to form lammelopodia. In fact, actin staining of PKR KO and WT MEFs indicate that KO cells contain less than one third of the amount of filamentous actin as WT cells. Given that knockdown of gelsolin inhibits virus uptake, the suppression of gelsolin activity by PKR represents a novel antiviral mechanism [143].

*Caspase-11 and caspase-1*: The Yuan lab has identified two actin-associated caspase-11 interacting proteins, flightless and actin interacting protein 1 (Aip1). Flightless is an actin-capping protein and a member of the gelsolin superfamily. It localizes caspase-11 to the leading edge of migrating cells and to insoluble cell fractions. It also binds to and inhibits caspase-1 activation and subsequent IL-1β maturation. Analysis of flightless cleavage products indicates that it may inhibit caspase-1 activity by serving as a pseudosubstrate for its catalytic activity. This inhibition of caspase-1 and relocalization of caspase-11 by flightless could suppress pro-IL-1β processing to the secreted form, thus dysregulating the inflammatory response [141,150,151]. Aip1 activates cofilin-mediated actin depolymerization. Binding to caspase-11 enhances this polymerization by a mechanism independent of caspase activity and leads to enhanced migration during inflammation [144].

*IFITM1*: Interferon-induced transmembrane protein 1 (IFITM1) is one of hundreds of IFN-stimulated genes. In hepatocytes, it was found to accumulate in tight junctions in HCV-infected patients undergoing IFN therapy. It bound to the tight junction proteins occludin, claudin-1, and ZO-1, and appeared to enhance the interaction between the HCV coreceptors occludin and CD81, yet inhibited virus entry. It is proposed that this disruption in entry results from the altered coordination of coreceptor interactions and complex formation, however this mechanism requires further investigation [146].

*ADAP2*: ADAP2 (ADP-ribosylation factor (Arf) GTPase-activating protein (GAP) with dual pleckstrin homology domains 2) is an IFN-inducible, GTPase-activating protein for Arf6. It associates with and can control actin dynamics, as well as induce membrane ruffling and macropinocytosis, leading to the formation of ADAP2-associated vesicles. These vesicles are also positive for Rab8a (recycling endosomes) and lysosomal-associated membrane protein 1 (LAMP1; lysosomes). ADAP2 expression shifts the endocytic association of Dengue virus and vesicular stomatitis virus (VSV) into these ADAP2-positive vesicles instead of Rab5 and Rab7 endosomes. This not only inhibits their entry into the cell but also likely delivers the viruses directly to degradative lysosomes [145].

## 7. A Network of Innate Immune Mediators Link Pathogen Sensing to Host Response via Cytoskeletal Associations: Emerging Model and Outstanding Questions

The emerging model of pathogen-induced actin alteration as a sensor and regulator of immune activation (Figure 5) involves the contribution of a diverse profile of cytoskeletal and innate immune components (Table 1). Interestingly, functional roles for cytoskeletal proteins in the innate immune response appears to occur primarily through an intersection with RLR and inflammasome pathways, whereas few TLR signaling components have been identified. Whether this is a fundamental difference between cytosolic receptors and membrane receptors, or if TLR interactors simply have yet to be found, is still to be determined.

If these interactions are truly beginning to define a regulatory mechanism for innate immune activity, it is interesting to speculate as to why it may have developed. Viruses and bacteria manipulate the cytoskeleton throughout the course of infection. Bacteria utilize actin polymerization for many purposes such as forming pedestals to mediate attachment and invasion, generating actin tails for disseminating throughout the cytosol, or to mediate intercellular spread [133]. Viruses remodel actin for almost every step of replication including invasion, migration to the nucleus, assembly, exocytosis, and budding [134]. These are all conserved and universal activities that are unlikely to be lost through adaptation, and therefore make pathogen-induced cytoskeletal perturbations good targets for innate immune activation. Most of these interactions localize normally cytosolic innate signaling molecules to the cytoskeleton, particularly to the cell membrane or tight junctions. Several viruses, such as HCV and Coxsackievirus, bind to receptors located at tight junctions, making this site an ideal location for innate immune activators [152,153]. Similarly, RNase-L, PKR, RIG-I, and NOD2 are all viral RNA sensors and proximity to viral entry may provide a kinetic advantage to the cell. In addition, RIG-I, RNase-L, and PKR have been found to associate with antiviral stress granules (avSG) that form after virus infection and contain viral RNA. Formation of these complexes is necessary for IFNβ induction in response to multiple stimuli [154,155]. avSGs are related to stress granules which form during cellular stress from inhibited translation and are linked to the cytoskeleton. If avSGs are also associated with the cellular structure, this may mean that RIG-I, PKR, and RNase-L either require their interaction with the cytoskeleton in order to be incorporated into avSGs or to control them. Indeed, RIG-I forms punctate domains when cells are treated with cytD to inhibit actin polymerization, however it is unknown whether these are avSGs. An alternative function of this association between members of the innate immune response and the cytoskeleton may be to sequester these proteins to prevent unintended activation. Incidental overexpression of IFN would lead to the needless upregulation of hundreds of genes and create an unnecessary heightened antiviral state in surrounding tissue. Similarly, RNase-L and PKR are both proapoptotic and require tight regulation to prevent accidental cell death [3].

The question that ultimately arises, however, is how does the cell distinguish between pathogen-induced cytoskeletal remodeling and that due to physiologic cellular processes? Inflammasome activation requires a priming step to transcriptionally induce IL-1β and IL-18 which indicates that an authentic PAMP is required to avoid spurious activation. The priming signal is typically initiated through TLR engagement. This upstream role may explain why TLR signaling molecules that are activated by cytoskeletal perturbations have not yet been identified [156]. RLR activation does not need a secondary stimulus and therefore may require an alternative mechanism for differentiating between friendly and foreign actin rearrangement. Collectively, this emerging area of study still has many discoveries to be made and questions to answer. Recent findings have shown that RNase-L is not only a member of this new class of host defense mechanisms, but may do so on multiple levels through its interactions with filamin A, IQGAP, and LNX. Despite over 40 years of research, this endoribonuclease still has many lessons to offer and may be a key to helping elucidate the link between cytoskeletal integrity and host defense.
